# Discrimination and adherence in a cross-sectional study of Latino sexual minority men with HIV: Coping with discrimination as a mediator and coping self-efficacy as a moderator

**DOI:** 10.1007/s10865-023-00426-6

**Published:** 2023-07-01

**Authors:** Joanna L. Barreras, Laura M. Bogart, Sarah MacCarthy, David J. Klein, David W. Pantalone

**Affiliations:** 1 School of Social Work, California State University Long Beach, 1250 Bellflower Boulevard, Long Beach, CA 90840 USA; 2 Bienestar Human Services, Inc, 5326 East Beverly Blvd, Los Angeles, CA 90022 USA; 3 RAND Corporation, 1776 Main Street, Santa Monica, CA 90407 USA; 4 Department of Health Behavior, University of Alabama, 1665 University Boulevard, Birmingham, AL 35294 USA; 5 Department of Psychology, University of Massachusetts Boston, 100 Morrissey Boulevard, Boston, MA 02125 USA; 6 The Fenway Institute, Fenway Health, 1340 Boylston Street, Boston, MA 02215 USA

**Keywords:** Adherence, Antiretroviral therapy, Coping, Intersectional stigma and discrimination, Latino/x/e, HIV/AIDS, Sexual minority men

## Abstract

Discrimination is associated with antiretroviral therapy non-adherence and reduced well-being among people with HIV. We examined the potential for coping to mediate the associations between intersectional discrimination and non-adherence and coping self-efficacy (confidence in one’s ability to cope with discrimination) as a moderator that may buffer the negative effects of discrimination on non-adherence in a cross-sectional convenience sample of 82 Latino sexual minority men with HIV. In bivariate linear regressions, discrimination targeting Latino ethnic origin, undocumented residency status, and sexual orientation were each significantly associated with lower self-reported antiretroviral therapy non-adherence (percentage of prescribed doses taken in the last month) and greater use of disengagement coping (denial, substance use, venting, self-blame, behavioral disengagement). Associations between discrimination targeting Latino ethnicity and non-adherence, and discrimination targeting undocumented residency status and non-adherence, were each mediated by disengagement coping responses. Moderation analyses highlighted significant discrimination by coping self-efficacy interaction effects—both coping self-efficacy for problem solving and stopping unpleasant emotions/thoughts each moderated the associations between Latino discrimination and adherence, between undocumented residency status discrimination and adherence, and between HIV discrimination and adherence. Coping self-efficacy for getting social support moderated the association between undocumented residency status discrimination and adherence. Further, the interaction coefficients across models indicated that the negative effects of discrimination on adherence were attenuated at higher levels of coping self-efficacy. Findings highlight the need for structural interventions that reduce—and ultimately eliminate—discrimination, and interventions that address the harmful effects of discrimination and adherence improvement interventions to enhance coping skills among people faced with intersectional discrimination.

## Introduction

In the United States, Latino sexual minority men are disproportionately impacted by HIV. In 2019, Latino gay and bisexual men comprised 22% of the 36,801 new HIV diagnoses (Centers for Disease Control and Prevention [CDC], 2019). Moreover, among newly diagnosed gay and bisexual men, 32% were Latino compared to 25% non-Hispanic White (CDC, 2019). These HIV-related inequities are thought to be rooted in intersectional discrimination, a concept originating in Black feminist scholarship that refers to interlocking forms of structural oppression, including racism, sexism, and heterosexism (Algarin et al., 2019; Berger, 2010; Bowleg, 2013; Crenshaw, 1989; Layland et al., 2020; Logie et al., 2018; Reisen et al., 2013). Intersectional discrimination includes harmful experiences that occur for an individual on the basis of multiple oppressed identities (e.g., being both gay and Latino).

Studies have documented associations between discrimination and a range of general (e.g., poor psychological and physical functioning) and HIV-specific health indicators (e.g., antiretroviral therapy non-adherence, medication side effects) in sexual minority men with HIV (Bogart et al., 2010, 2013a, b; Galvan et al., 2017; LaVeist et al., 2000; Wagner et al., 2012). For example, results from a quantitative, cross-sectional survey study with a majority Latino sample of sexual minority adults with HIV found that discrimination (operationalized as general mistreatment, not specific to any characteristics or identity) was positively associated with depressive symptoms (Tabler et al., 2021). With respect to HIV-related health indicators, results from another quantitative cross-sectional study indicated significant combined effects of Latino ethnicity, sexual orientation, and HIV discrimination on greater medication side effect severity and AIDS symptoms (Bogart et al., 2013a, b).

Studies also have begun to illuminate associations between discrimination and HIV care retention (Martinez, 2019; Ortiz-Sánchez et al., 2017; Varas-Díaz et al., 2019) for sexual minority men with HIV. For example, results from a qualitative, cross-sectional, in-depth interview study showed both individual-level intersectional characteristics (e.g., being Latino, gay, and undocumented) and structural-level experiences with intersectional discrimination (e.g., in healthcare, punitive immigration policies, institutionalized homophobia) were associated with interruptions in HIV care, including lower care engagement and antiretroviral therapy non-adherence (Barrington et al., 2021). Another qualitative in-depth interview study of Latino immigrant and migrant adults with HIV indicated that discrimination by friends and family was a barrier to consistent HIV care, and receiving family and friend support promoted HIV care engagement (Levison et al., 2017).

In the present study, we drew on theoretical frameworks of stress, discrimination, coping, and health (Lazarus & Folkman, 1987; Weaver et al., 2005; Williams & Mohammed, 2013) to understand associations among discrimination, coping, and adherence to HIV treatment among Latino sexual minority men. Specifically, we conceptualized coping with discrimination as a mediator, or mechanism, that could explain the effects of discrimination on non-adherence. We extended Weaver et al.’s (2005) stress and coping model of adherence to consider discrimination as a form of stress, consistent with Williams and Mohammed (2013)’s model of discrimination and health, which posits that coping is a response to discrimination (i.e., a mediator that temporally follows discrimination) that affects stress responses and consequent health outcomes. For example, in a quantitative cross-sectional survey study, coping was shown to mediate the effect of discrimination targeting ethnicity on general health behaviors among Latino immigrant gay men with HIV (Bianchi et al., 2004).

In contrast, we conceptualized coping self-efficacy as a moderator that may buffer the negative effects of discrimination on non-adherence, consistent with Lazarus and Folkman’s transactional theory of coping. Specifically, coping self-efficacy (i.e., confidence one has in the ability to use existing coping skills in vivo) may be conceptualized as a generalized belief, or resilience resource, that is not directly tied to an individual stressor, such as a discrimination experience. Thus, as individuals use effective coping strategies repeatedly over time, they may build self-confidence for generalizing coping strategies across discrimination experiences. For example, quantitative survey research with people with HIV has demonstrated that coping self-efficacy moderates the impact of HIV-related discrimination on self-reported health status (Prati & Pietrantoni, 2016).

Building on prior theories and findings discussed above, we tested the hypotheses that coping with discrimination would mediate associations between discrimination and antiretroviral therapy non-adherence, and that coping self-efficacy would moderate such associations in a cross-sectional survey of Latino sexual minority men with HIV, a population affected strongly by intersectional discrimination related to ethnicity, immigration status, sexual minority orientation, and HIV. Although the present study’s primary focus was on coping, we recognize the overarching need to alleviate and prevent discrimination at the structural-societal level, in tandem with understanding how to improve mental health and coping to reduce the impact of discrimination at the individual level.

## Method

### Study setting

This study was conducted in Los Angeles County, home to one of the largest HIV epidemics in the United States. In Los Angeles County, by the end of 2021, Latino men represented 41% of all people with diagnosed HIV; further, in 2020, compared to non-Latine White individuals, Latine individuals had higher diagnosis and death rates from HIV and AIDS, and Latine individuals with HIV were less likely to be adherent to antiretroviral therapy and virally suppressed (Division of HIV and STD Programs, Department of Public Health, County of Los Angeles, 2022).

We used community-based participatory research methods (Minkler & Wallerstein, 2011), conducting the study in the context of an established community-academic partnership with one of Los Angeles County’s largest HIV services community-based organizations (CBOs) serving primarily Latino sexual and gender minority adults. Specifically, the CBO’s community advisory board (CAB) actively collaborated with the research team, upholding shared-decision making and providing their expertise and support during all aspects of the research: before, during, and after the study. Moreover, the CAB is one of the CBO’s strongest assets, comprised of community members who are clients of the CBO and are considered the voice of the community and study population.

### Participants

This study used baseline data from a pilot randomized controlled trial of an intervention to strengthen coping with discrimination and improve antiretroviral therapy adherence among Latino sexual minority men with HIV (Bogart et al., 2021). From March, 2018 until February, 2019, we recruited a convenience sample via study fliers (hard copies and social media), staff and client presentations at our community partner organization, and word-of-mouth. Participants were eligible if they self-identified as Latino; were at least 18 years of age; were living with HIV; were cisgender men (i.e., were assigned male sex at birth and identified as male gender); ever had sex with another man; and either (a) were not on antiretroviral therapy, (b) were on antiretroviral therapy but missed a dose in the last month, or (c) had fewer than two HIV care visits in the last year. Individuals were ineligible if they identified their gender identity as transgender, as the intervention being tested was focused narrowly on sexual orientation and not gender identity-related discrimination or other concerns specific to transgender populations (e.g., gender identity concealment).

Of 92 individuals screened for eligibility, nine were ineligible and one eligible participant declined to enroll. Thus, we enrolled 82 participants. Participants completed the baseline survey in Spanish using an audio computer-assisted self-interview with support from a research assistant. Participants received a $40 gift card for completing the survey. All study procedures were approved by the RAND Corporation Human Subjects Protection Committee and Los Angeles County Public Health, Ambulatory Care Network and Health Services Administration Institutional Review Board. We obtained written informed consent from all participants. Note that the sample size was based on a power analysis that determined the number of participants needed to detect a medium-to-large pilot intervention effect; a power analysis was not conducted a priori for the present analysis.

### Measures

#### Sociodemographic characteristics

From all participants, we collected data on age, household income, sexual orientation, education level, employment status, housing status, relationship status, residency status, and length of time in the U.S. In addition, we asked participants the month and year they received their HIV diagnosis (Table 1).

**Table 1:** Descriptive Statistics and Associations of Socio-Demographic Variables with Antiretroviral Therapy Adherence in a Sample of 82 Latino Sexual Minority Men with HIV

Variable	M (SD) or n (%)	Bivariate Association with Self-Reported Adherence (% of Doses Taken, Past Month) b (SE)	p-value
Socio-Demographic Characteristic			
Age	52.0 (13.1)	0.02 (0.15)	0.91
Highest Education Level ^a^		-5.71 (3.76)	0.13
None	2 (2.4%)		
+ Less than high school	34 (41.5%)		
High school diploma or GED	24 (29.3%)		
Associate degree (junior or 2- year college)	10 (12.2%)		
Bachelor’s or 4-year college degree	11 (13.4%)		
Graduate degree (M.A., Ph.D. or professional degree)	1 (1.2%)		
Employment Status ^b^		-6.57 (4.28)	0.13
Working full-time	11 (13.4%)		
Working part-time	10 (12.2%)		
Unemployed and looking for work	11 (13.4%)		
Disabled and not working	35 (42.7%)		
Not working and not looking for work	5 (6.1%)		
Retired	8 (9.8%)		
None of the above	2 (2.4%)		
Housing Arrangement past 12 months (not mutually exclusive) ^c^		-5.37 (4.23)	0.21
Renting home or apartment	23 (28.1%)		
Living in home/apartment owned by you or someone else in household	36 (43.9%)		
Public subsidized housing	18 (22.0%)		
Unstable housing (street, shelter, etc.)	7 (8.5%)		
Household Income in Past 12 Months ^d^		0.03 (4.31)	0.99
Less than $5,000	23 (29.1%)		
$5,000 − $11,999	31 (39.2%)		
$12,000 − $15,999	14 (17.7%)		
$16,000 − $24,999	6 (7.6%)		
$25,000 − $34,999	2 (2.5%)		
$35,000 and greater	3 (3.8%)		
Married or in a committed relationship	16 (19.5%)	4.16 (4.76)	0.38
Sexual Identity ^e^		0.54 (4.67)	0.91
More straight than gay	4 (4.9%)		
Equally straight and gay	4 (4.9%)		
More gay than straight	4 (4.9%)		
Mostly gay	3 (3.7%)		
Exclusively gay	65 (79.3%)		
Do not identify with any orientation	2 (2.4%)		
Residency Status ^f^		7.25 (4.07)	0.08
U.S. citizen	24 (29.6%)		
Permanent resident	17 (21.0%)		
Undocumented Status	25 (30.9%)		
Temporary visa	15 (18.5%)		
**HIV-Related Characteristics**			
Self-reported adherence, past month	90.8 (17.0)	N/A	
Time since HIV diagnosis (years)	15.9 (9.6)	0.00 (0.21)	0.99

Notes: For estimating correlations with adherence, sample characteristics were dichotomized as indicators for: (a) Less than high school education; (b) Working (full-time or part-time); (c) Having lived anywhere but own home/apartment past year; (d) Less than $5K income; (e) Exclusively gay (note: no one endorsed the “exclusively straight” or “mostly straight” response options and thus they are not shown in the table); and (f) Undocumented. Only income (n = 3), residency status (n = 1), and time since HIV diagnosis (n = 5) had missing values, of all variables listed in the table

#### Intersectional discrimination

The Multiple Discrimination Scale (MDS) was used to examine discrimination experiences (i.e., enacted stigma, or concrete behavioral expressions of prejudice) in the last 12 months (Bogart et al., 2010). Items on the MDS ask participants if they have experienced any of 13 different types of discrimination events with respect to ethnic background (“because you are Latino”; α = .86), undocumented U.S. residency status (“because someone thought or knew that you were not a legal U.S. resident” α = .85), sexual minority orientation (“because someone thought you were gay”; α = .84), and HIV-positive status (“because you are HIV-positive”; α = .82). Response options were “yes,” “no,” and “not applicable.” Items covered various expressions of discrimination, including violent acts (e.g., “physically assaulted or beaten up”), institutional discrimination (e.g., “denied a job or lost a job’’), and interpersonal discrimination (e.g., “treated with hostility or coldness by strangers”). We calculated a summary score for each subscale, with a range of 0 to 10; higher scores represented more discrimination experiences.

#### Coping with discrimination

We adapted the Brief COPE (Carver, 1997), adding items based on previous formative research with the population (Bogart et al., 2021; MacCarthy et al., 2021), that asked participants to “indicate the extent you do what the item says when you are faced with discrimination.” Based on recommendations to tailor Brief COPE subscales to match study goals and theoretical frameworks (Carver, 1997; Compas et al., 2001; Solberg et al., 2022), we created two subscales: an “engagement coping with discrimination subscale,” i.e., coping responses oriented toward the source of stress or one’s emotions or thoughts (α = 0.82; 19 items), defined as the mean of items related to active coping, use of emotional support, use of instrumental support, positive reframing, planning/strategic avoidance, humor, acceptance, and religion; and a “disengagement coping with discrimination subscale,” i.e., coping responses oriented away from the stressor or one’s emotions or thoughts (α = 0.71; 12 items), operationalized as the mean of items related to denial, substance use, venting, self-blame, behavioral disengagement, and self-distraction. Response options were 1 = “I haven’t been doing this at all,” 2 = “I’ve been doing this a little bit,” 3 = “I’ve been doing this a medium amount,” 4 = “I’ve been doing this a lot,” and “not applicable.”

#### Coping self-efficacy

To assess coping self-efficacy, we used the 26-item Coping Self Efficacy Scale (CSES; Chesney et al., 2006), in which participants are asked, “When things aren’t going well for you, how confident are you that you can do the following?” and then rate their confidence that they could perform behaviors important to effective coping on an 11-point Likert-scale (0 = “cannot do at all,” 5 = “moderately certain can do,” to 10 = “certain can do”). The CSES has three subscales: being problem-focused (α = 0.81), being able to stop unpleasant emotions and thoughts (α = 0.66), and obtaining support from family and friends (α = 0.79).

#### Self-reported antiretroviral therapy adherence

Participants were asked to estimate the percentage of prescribed antiretroviral therapy doses that they took in the past month using a visual analogue scale (continuous outcome, 0-100%) (Simoni et al., 2006).

### Statistical analysis

We calculated descriptive statistics for all variables. We used Pearson correlations to assess the associations between antiretroviral therapy adherence and potential socio-demographic covariates for subsequent analyses. None of these correlations were statistically significant (at *p* < 0.05); thus, we did not include any covariates in the mediation or moderation analyses. Moreover, none of the variables involved in the mediation or moderation analyses had any missing values.

To test for mediation and moderation, we used a series of linear regressions with ordinary least squares estimation. For mediation, the regressions consisted of: (1) the mediator (coping) with the independent variable (discrimination); (2) the mediator (coping) with the dependent variable (continuous antiretroviral therapy adherence); and (3) the dependent variable (continuous antiretroviral therapy adherence) with the independent variable (discrimination) and the mediator (coping). To assess whether a significant portion of the effect of each discrimination variable on antiretroviral therapy adherence was accounted for by coping with discrimination, we used PROC CAUSALMED (SAS v9.4), a causal mediation approach which is robust to non-linear and interaction associations, unlike statistical mediation, although both assume causality (Fairchild & McDaniel, 2017; Lee et al., 2019). PROC CAUSALMED combines the results of the two regression models to estimate direct and indirect effects using a bootstrapping approach, resulting in a 95% confidence interval, with the absence of zero indicating significance at *p* < 0.05 (Preacher & Hayes, 2008). A total of eight sets of regression models were used to test for mediation, for the four types of discrimination by the two types of coping with discrimination, to test the hypothesis that coping is an underlying mechanism that helps to explain the association between discrimination and non-adherence (i.e., “mediation for explanation”) (Fairchild & McDaniel, 2017).

To test for moderation, each regression predicting continuous antiretroviral therapy adherence included the main effect of discrimination, the main effect of coping self-efficacy, and the interaction between discrimination and coping self-efficacy. A total of twelve regression models were used to test for moderation, for each of the four types of discrimination × each of the three types of coping self-efficacy.

## Results

### Participant characteristics

Table 1 shows the sample’s descriptive statistics, as well as associations of socio-demographic and coping variables with antiretroviral therapy adherence. Participants had a mean age of 52 years, about one-fifth were married or in a committed relationship, and about three-quarters were not working full- or part-time. Nearly 9% were unstably housed and over two-thirds had very low incomes (of <$12,000 annually). Nearly all (88%) identified as “exclusively gay,” “mostly gay,” or “more gay than straight.” Approximately one-third were undocumented and almost one-fifth had a temporary visa. Self-reported antiretroviral therapy adherence was high on average (M = 90.8% of doses taken; [SD = 17.0]) and the mean length of time since diagnosis was about 16 years (SD = 9.6).

### Bivariate associations among discrimination, coping with discrimination, coping self-efficacy, and antiretroviral therapy adherence

Bivariate linear regressions indicated that higher disengagement coping was associated with lower antiretroviral therapy adherence, and all three coping self-efficacy subscales (i.e., confidence in one’s ability to be problem-focused, to stop unpleasant emotions and thoughts, and to get support from family and friends) were associated with better antiretroviral therapy adherence (Table 2). Latino, undocumented residency status, and sexual minority orientation discrimination were all significantly associated with lower antiretroviral therapy adherence (Table 2) and greater use of disengagement coping (Table 3).

**Table 2:** Bivariate Linear Regression Coefficients for Associations of Perceived Discrimination, Coping, and Coping Self-efficacy with Self-Reported Antiretroviral Therapy Adherence

	M (SD)	Self-Reported Adherence (% of Doses Taken, Past Month) b (SE)	p-value
Discrimination Type (Multiple Discrimination Scale)			
Latino ethnicity	2.02 (2.82)	-1.57 (0.65)	0.02
Undocumented residency status	1.77 (2.61)	-1.43 (0.71)	0.049
Sexual minority orientation	2.10 (2.72)	-1.48 (0.68)	0.03
HIV positive serostatus	1.45 (2.24)	-1.23 (0.84)	0.15
Coping (Adapted Brief COPE)			
Engagement Coping	2.76 (0.49)	3.91 (3.90)	0.32
Disengagement Coping	2.15 (0.46)	-8.99 (3.99)	0.03
Coping Self-Efficacy (CSES)			
Problem-focused	8.11 (1.59)	3.58 (1.13)	0.002
Stopping unpleasant emotions/thoughts	8.51 (1.46)	3.85 (1.23)	0.002
Getting support friends/family	7.86 (2.14)	2.23 (0.85)	0.01

**Table 3:** Bivariate Linear Regression Coefficients for Associations of Perceived Discrimination with Coping Responses to Discrimination

	Engagement Coping ^a^ b (SE), p	Disengagement Coping ^a^ b (SE), p
Latino Ethnicity Discrimination	0.00 (0.02), p = 0.92	0.04 (0.02), p = 0.03
US Residency Status Discrimination	0.00 (0.02), p = 0.90	0.04 (0.02), p = 0.02
Sexual Orientation Discrimination	0.01 (0.02), p = 0.69	0.04 (0.02), p = 0.03
HIV-Serostatus Discrimination	0.02 (0.02), p = 0.43	0.02 (0.02), p = 0.36

^a^ Assessed with Brief COPE

### Coping responses to discrimination as mediators between intersectional discrimination and antiretroviral therapy adherence

Based on the significant bivariate tests, we tested disengagement coping as a mediator in the associations between discrimination and antiretroviral therapy adherence (Table 4). Disengagement coping was found to mediate associations of Latino and undocumented residency status discrimination with antiretroviral therapy adherence. Specifically, the association between Latino discrimination and adherence was non-significant when disengagement coping was added to the regression model; there was a significant indirect effect of this association via disengagement coping, explaining 17.8% of the variance; and the bootstrap-based confidence interval for the indirect effect did not include zero. Similarly, the direct association between discrimination targeting residency status (being undocumented) and antiretroviral therapy adherence was not significant when disengagement coping was added to the regression model; the indirect effect of this association via disengagement coping was significant, explaining 23.4% of the variance; and the bootstrap-based confidence interval for the indirect effect did not include zero.

**Table 4:** Ordinary Least Squares Linear Regression Models Testing Coping with Discrimination as a Mediator of the Association between Discrimination and Adherence

Variables	Specific and total indirect effects, direct effects, and total effects with ART Adherence	*Estimate*	*S.E.*	*95% bootstrap CI*	*Percentage of Variance Mediated*
Latino Discrimination	Indirect effect through disengagement coping	-0.28	0.23	-1.05, -0.002	17.8%
	Direct effect	-1.29	1.08	-3.73, 0.61	
	Total effect	-1.57	1.05	-3.82, 0.33	
Residency Status Discrimination	Indirect effect through disengagement coping	-0.33	0.25	-1.14, -0.03	23.4%
	Direct effect	-1.09	1.15	-3.87, 0.68	
	Total effect	-1.43	1.12	-4.08, 0.39	

S.E. = bootstrap standard error (5,000 replications)

### Coping self-efficacy as a moderator of the association between intersectional discrimination and antiretroviral therapy adherence

Results of the regression moderation analyses indicated significant discrimination by coping self-efficacy interaction effects in seven of the twelve models (Table 5): coping self-efficacy for both problem solving and stopping unpleasant emotions/thoughts moderated the associations between Latino discrimination and adherence, between undocumented residency status discrimination and adherence, and between HIV discrimination and adherence. Moreover, coping self-efficacy for getting social support moderated the association between undocumented residency status discrimination and adherence. As indicated by the interaction coefficients, across models, the negative effects of discrimination on adherence were attenuated when coping self-efficacy was high, consistent with hypotheses.

**Table 5:** Ordinary Least Squares Linear Regression Models Testing Coping Self-Efficacy (CSE) as a Moderator of the Association between Discrimination and Adherence

	b	SE	p
**Model 1: Latino discrimination, problem solving coping self-efficacy**			
Latino discrimination main effect	-10.70	2.29	< 0.001
Problem solving CSE main effect	-0.10	1.28	0.94
Latino discrimination X Problem solving CSE interaction	1.27	0.30	< 0.001
**Model 2: HIV discrimination, problem solving coping self-efficacy**			
HIV discrimination main effect	-17.71	4.11	< 0.001
Problem solving CSE main effect	-0.20	1.37	0.89
HIV discrimination X Problem solving CSE interaction	2.05	0.50	< 0.001
**Model 3: Undocumented residency discrimination, problem solving coping self-efficacy**			
Undocumented residency discrimination main effect	-11.34	2.58	< 0.001
Problem solving CSE main effect	0.13	1.30	0.92
Undocumented residency discrimination X Problem solving CSE interaction	1.37	0.33	< 0.001
**Model 4: Latino discrimination, stopping unpleasant emotions/thoughts oping self-efficacy**			
Latino discrimination main effect	-9.36	2.70	< 0.001
Stopping unpleasant emotions/thoughts coping self-efficacy main effect	-0.13	1.62	0.93
Latino discrimination X stopping unpleasant emotions/thoughts coping self-efficacy interaction	1.02	0.32	0.002
**Model 5: HIV discrimination, stopping unpleasant emotions/thoughts coping self-efficacy**			
HIV discrimination main effect	-21.49	5.41	< 0.001
Stopping unpleasant emotions/thoughts CSE main effect	0.00	1.51	0.99
HIV discrimination X stopping unpleasant emotions/thoughts CSE interaction	2.40	0.63	< 0.001
**Model 6: Undocumented residency discrimination, stopping unpleasant emotions/thoughts coping self-efficacy**			
Undocumented residency discrimination main effect	-7.77	2.76	0.006
Stopping unpleasant emotions/thoughts CSE main effect	0.82	1.60	0.61
Undocumented residency discrimination X stopping unpleasant emotions/thoughts CSE interaction	0.85	0.33	0.01
**Model 7: Undocumented residency discrimination, getting support coping self-efficacy**			
Undocumented residency discrimination main effect	-6.09	2.49	0.02
Getting support CSE main effect	1.05	0.97	0.28
Undocumented residency discrimination X getting support CSE interaction	0.68	0.34	0.047

## Discussion

The present study extends prior research and provides insights for potential individual-level intervention levers to improve antiretroviral therapy adherence for vulnerable populations of people with HIV, including Latino sexual minority men. Consistent with prior research documenting the negative consequence of discrimination on health in general (Pascoe & Smart Richman, 2009), and specifically on HIV-related health indicators (Bogart et al., 2010, 2013a, b), we found that Latino, undocumented status, and sexual minority orientation discrimination were significantly and negatively associated with self-reported antiretroviral therapy adherence. Moreover, we found that the association between experiencing discrimination and non-adherence can be explained at least in part by disengagement coping strategies. Also consistent with prior research (Prati & Pietrantoni, 2016), we found that greater confidence in one’s ability to cope with challenges—by focusing on problem solving, stopping unpleasant thoughts and emotions, and seeking support from family and friends—buffered the associations between discrimination and non-adherence to self-reported antiretroviral therapy. Overall, these results provide further support for the theories of stress, coping, and discrimination that underpinned our research (Lazarus & Folkman, 1987; Weaver et al., 2005; Williams & Mohammed, 2013), and can help to generate novel insights about how and under what conditions discrimination may affect health. Taken together, our findings underscore the need to reduce disengagement coping strategy use in combination with increasing confidence and skills in using engagement coping strategies to bolster social support, problem solve, and stop unpleasant thoughts and emotions.

Our research highlights the need for structural interventions, beyond the individual level, that reduce and ultimately end intersectional discrimination in the long-term. In the short-term, individual-level interventions that address the harmful effects of discrimination are needed to provide psychoeducation about how to recognize use of ineffective disengagement coping strategies, as well as to build coping self-efficacy and increase confidence to implement such skills. Prior qualitative research on coping among people affected by intersectional discrimination has found that Latino sexual minority men with HIV often employ effective coping strategies, including strategic avoidance of discriminatory environments or people, social support, self-advocacy, and external attribution (i.e., placing blame on the perpetrator; (MacCarthy et al., 2021). To counter discrimination’s negative effects, our findings suggest a need for interventions that promote effective engagement coping strategies that leverage innate resiliencies, as well as confidence in one’s ability to leverage innate resiliencies, rather than teaching a new toolkit of skills. Further, interventions should be developed and disseminated that instill skills regarding how and in which situations to apply innate effective coping skills, to ensure that Latino sexual minority men have the confidence to put coping skills into practice and are able to obtain support from friends and family to do so. Some prior cognitive behavior therapy-based intervention work (e.g., cognitive behavioral stress management interventions) has shown success in improving coping, coping self-efficacy, and/or adherence among people with HIV (Brown & Vanable, 2008; Carrico et al., 2006; Jones et al., 2007). For example, results from a randomized controlled trial testing a group-based intervention for sexual minority men with HIV found that significant increases in coping self-efficacy mediated improvements in anxiety, stress, and well-being (Chesney et al., 2003).

The present study results also suggest ways that coping interventions can be tailored for individuals who experience intersectional discrimination. Only a few studies to our knowledge have specifically developed and tested interventions for coping with the stress of discrimination in particular; these interventions have been found to improve antiretroviral therapy adherence and coping among Latino and Black sexual minority men with HIV in pilot randomized controlled trials (Bogart et al., 2018, 2020, 2021). Intervention developers also could consider integrating a social support assessment so that individuals with insufficient social support could be linked to peer support groups or other social opportunities for individuals faced with similar intersectional characteristics (e.g., volunteering in organizations for sexual minority people of color).

There are several limitations to consider when interpreting our results. First, although we used a validated measure of adherence, self-reported antiretroviral therapy adherence tends to be an overestimate of true adherence (Stirratt et al., 2015); however, we do not expect that the observed associations between discrimination, coping, and antiretroviral therapy adherence, would be different for those with lower adherence, as prior research on discrimination and antiretroviral therapy adherence using more objective adherence measures has shown similar associations (Bogart et al., 2010). Second, generalizability is limited, as this study was based in Los Angeles, California, and a small convenience sample of adult sexual minority male participants were primarily recruited from one Latine HIV service organization and were mostly immigrants from Mexico. Third, this study used a cross-sectional design resulting in correlational, non-experimental data to test for mediation, which does not allow for causal temporal inferences; thus, reverse causation, such as adherence predicting coping and discrimination, is possible. However, the present study’s firm basis in stress and coping and discrimination theories, as well as prior research (in which mental health issues have been found to result from, and not predict, discrimination) (English et al., 2014; Schulz et al., 2006), suggests that reverse causality is unlikely. Our item wording also lends credence to the presumption of temporal ordering, as participants were asked about coping responses specifically in relation to discrimination. Moreover, unmeasured confounding could have biased the indirect and direct effect estimates in the mediation model (Fairchild & McDaniel, 2017; Lee et al., 2019). Fourth, our sample was relatively small and, thus, the analysis was likely underpowered—which could explain why some but not all associations tested were non-significant. Fourth, the discrimination scale that we used, the MDS, assesses discrimination separately for each marginalized characteristic, rather than in combination, as an intersectional construct. Future research is needed to understand the effects of intersectional discrimination on adherence for HIV, as well as other conditions, and related coping strategies.

## Conclusion

The deleterious effects of discrimination on health are well-documented. Our study suggests that coping could be a potentially powerful lever to minimize the negative effects of intersectional discrimination on antiretroviral therapy adherence in Latino sexual minority men with HIV. In the future, programs, policies, and research that aims to address health inequities faced by Latino sexual minority men with HIV should explore how to bolster existing coping strategies and people’s ability to implement them in ways that acknowledge and validate people’s whole selves across a range of identities. Ultimately, beyond the individual level, structural interventions are needed to eliminate discrimination. These structural interventions can include addressing discriminatory policies and change in societal institutions.

## Data Availability

Participants were not asked to provide consent for data sharing and thus individual-level data are not available.

## Code availability

The SAS code used for the analysis is available upon request to the second author at lbogart@rand.org (LMB).

## Declarations

### Clinical Trials Registration Number

NCT03432819
