# Correction to: Discrimination and adherence in a cross-sectional study of Latino sexual minority men with HIV: Coping with discrimination as a mediator and coping self-efficacy as a moderator

**DOI:** 10.1007/s10865-023-00449-z

**Published:** 2023-09-28

**Authors:** Joanna L. Barreras, Laura M. Bogart, Sarah MacCarthy, David J. Klein, David W. Pantalone

**Affiliations:** 1 School of Social Work, California State University Long Beach, 1250 Bellflower Boulevard, 90840 Long Beach, CA USA; 2 Bienestar Human Services, Inc, 5326 East Beverly Blvd, 90022 Los Angeles, CA USA; 3 RAND Corporation, 1776 Main Street, 90407 Santa Monica, CA USA; 4 Department of Health Behavior, University of Alabama, 1665 University Boulevard, 35294 Birmingham, AL USA; 5 Department of Psychology, University of Massachusetts Boston, 100 Morrissey Boulevard, 02125 Boston, MA USA; 6 The Fenway Institute, Fenway Health, 1340 Boylston Street, 02215 Boston, MA USA

**Correction to: Journal of Behavioral Medicine**

10.1007/s10865-023-00426-6

The article “Discrimination and adherence in a cross-sectional study of Latino sexual minority men with HIV: Coping with discrimination as a mediator and coping self-efficacy as a moderato” written by Joanna L. Barreras, Laura M. Bogart, Sarah MacCarthy, David J. Klein and David W. Pantalone was originally published electronically on the publisher’s internet portal on [1-July 2023] with the copyright “This is a U.S. Government work and not under copyright protection in the US; foreign copyright protection may apply 2023”. But it should have been “The RAND Corporation”

The original article has been corrected.

